# Peritraumatic Context and Long-Term Outcomes of Concussion

**DOI:** 10.1001/jamanetworkopen.2024.55622

**Published:** 2025-01-22

**Authors:** Emily J. Van Etten, Arielle R. Knight, Tristan A. Colaizzi, Jack Carbaugh, Alexandra Kenna, Catherine B. Fortier, William P. Milberg

**Affiliations:** 1 Translational Research Center for TBI and Stress Disorders, Veterans Affairs Boston Healthcare System, Boston, Massachusetts; 2 Department of Psychiatry, Harvard Medical School, Boston, Massachusetts; 3 Department of Psychiatry, Boston University Chobanian and Avedisian School of Medicine, Boston, Massachusetts; 4 Geriatric Research, Educational and Clinical Center, Veterans Affairs Boston Healthcare System, Boston, Massachusetts

## Abstract

**Importance:**

There has been a great deal of interest in mild traumatic brain injury (mTBI) and posttraumatic stress disorder (PTSD) and their association with one another, yet their interaction and subsequent associations with long-term outcomes remain poorly understood.

**Objective:**

To compare the long-term outcomes of mTBI that occurred in the context of psychological trauma (peritraumatic context) with mTBI that did not (nonperitraumatic context).

**Design, Setting, and Participants:**

This cohort study of post-9/11 US veterans used data from the Translational Research Center for Traumatic Brain Injury and Stress Disorders (TRACTS) study at the Veterans Affairs Boston Healthcare System, which began in 2009; the current study utilized data from baseline TRACTS visits conducted between 2009 and 2024. Data analysis occurred from January to October 2024.

**Exposures:**

Peritraumatic mTBI, nonperitraumatic mTBI, or no TBI.

**Main Outcomes and Measures:**

The primary outcomes were PTSD severity (measured by the Clinician-Administered PTSD Scale–4th edition), postconcussive symptoms (measured by the Neurobehavioral Symptom Inventory), and self-reported disability status (measured by the World Health Organization Disability Assessment Schedule II). Differences between groups were compared using analyses of covariance with least significant difference comparisons.

**Results:**

This sample of 567 post-9/11 veterans (mean [SD] age, 33.72 [9.29] years; 507 men [89.4%]; mean [SD] years of education, 14.19 [2.16]) included 183 individuals with no TBI, 189 individuals with nonperitraumatic mTBI, and 195 individuals with peritraumatic mTBI. Veterans with a history of peritraumatic mTBI had greater PTSD severity (*F*_2,552_ = 8.45; *P* < .001), postconcussive symptoms (*F*_2,533_ = 11.09; *P* < .001), and disability (*F*_2,527_ = 11.13; *P* < .001) than the nonperitraumatic mTBI and no TBI groups. Importantly, no significant differences in any outcome measure between nonperitraumatic mTBI and no TBI groups were observed.

**Conclusions and Relevance:**

This cohort study found that mTBI was only associated with long-term consequences when it co-occurred with a traumatic event exposure. This finding raises a novel hypothesis of the association of mTBI with PTSD, in which the acute biological and physiological outcomes of mTBI may be associated with temporarily scaffolding the formation of PTSD symptoms, which could enhance the production of long-term postconcussive symptoms and disability.

## Key Points

**Question:**

Are the long-term consequences of mild traumatic brain injury (mTBI) associated with the context in which it occurred?

**Findings:**

In this cohort study of 567 veterans, those with a history of peritraumatic mTBI had greater posttraumatic stress disorder severity, postconcussive symptoms, and disability than those with a history of nonperitraumatic mTBI and no TBI. No significant differences between nonperitraumatic mTBI and no TBI groups were observed.

**Meaning:**

These findings led to the development of a proposed framework that suggests that mTBIs sustained in traumatic contexts may create conditions that scaffold posttraumatic stress disorder symptom formation, inducing greater long-term disability and postconcussive symptoms.

## Teaser

This cohort study compares posttraumatic stress disorder (PTSD) severity, postconcussive symptoms, and disability status among US veterans with a history of peritraumatic and nonperitraumatic mild traumatic brain injury or no traumatic brain injury.

## Introduction

The high prevalence of mild traumatic brain injury (mTBI), often termed concussion, among post-9/11 US veterans^1,2,3^ has generated marked concern regarding the long-term physical, psychiatric, and functional outcomes of this condition. Despite increased attention and research on persistent mTBI-related long-term outcomes, the literature remains unresolved and contradictory, particularly regarding the association of common comorbidities with the same long-term outcomes. The current literature ranges widely, but can be divided into 3 broad analytic approaches: (1) studies that examine mTBI as an isolated predictor of enduring consequences without considering the confounding effects of prevalent comorbidities; (2) studies that look at mTBI as a predictor of various outcomes while controlling for those comorbidities as confounds; and (3) studies that treat mTBI categorically and use extant comorbidities as outcome measures.

The findings reflected in the first analytic approach often suggest mTBI is associated with long-lasting and/or progressive physical, cognitive, and/or functional difficulties.^4,5,6,7,8,9^ Studies using the second analytic approach find that deficits typically attributed to mTBI are almost fully accounted for by co-occurring conditions, particularly posttraumatic stress disorder (PTSD), with limited residual mTBI-related contributions.^10,11,12,13,14,15,16,17,18,19,20^ However, the findings of the third analytic approach seemingly contradict the second approach, and consistently demonstrate that the rate and severity of several psychiatric conditions, particularly PTSD, are greater in individuals with a history of mTBI than in those without.^1,2,21,22,23,24,25^ These studies then treat mTBI and PTSD as isolated categories, without considering their covariance, and show that individuals who have both a history of mTBI and PTSD display poorer outcomes than either condition alone, thus, arguing that mTBI is associated with psychiatric symptom formation and interacts with those comorbid conditions to increase likelihood of greater long-term consequences.^26,27,28,29^ Given their incompatible nature, these 3 analytic approaches are mutually exclusive—there cannot be both significant long-term outcomes of mTBI and no long-term outcomes of mTBI.

Unfortunately, none of these empirical approaches alone currently reconcile all the findings derived from the 3 approaches. These approaches may be contradictory because each typically considers mTBI and PTSD as isolated conditions that occur independently without acknowledging that oftentimes the circumstances that are associated with mTBI are also associated with psychological trauma.^25^ For example, mTBIs sustained during combat often occur at the same time and within the same context as a potential traumatic event (eg, improvised explosive device blasts). However, no studies to date have examined differences in outcomes between mTBIs that have co-occurred temporally with psychological trauma (peritraumatic context) vs mTBIs that occurred at distinct times and circumstances.

Comparing mTBIs sustained in a peritraumatic context with those that occurred in nonperitraumatic contexts may address a critical gap in both the empirical basis and theoretical explanatory approaches to understanding the long-term consequences of mTBI. Increasing evidence shows that biological and physiological responses during trauma are important predictors of PTSD symptom formation^30^ because these mechanisms can mediate how one processes traumatic stimuli. The co-occurrence of mTBI with traumatic events may temporarily disrupt the same cognitive, physiological, and neural coping mechanisms responsible for effectively processing traumatic experiences and protecting against the development of PTSD.^31^ Therefore, an mTBI may temporarily create the circumstances to allow for the formation of PTSD symptoms, which could lead to enduring deficits. To our knowledge, this study is the first to examine whether individuals who sustained a mTBI in a peritraumatic context would have greater long-term PTSD severity, postconcussive symptoms, and disability compared with individuals who sustained mTBIs in nonperitraumatic contexts and those with no TBI history.

## Method

### Participants

This cohort study was approved by the institutional review board of human studies research at the Veterans Affairs Boston Healthcare System (VABHS) and followed the Strengthening the Reporting of Observational Studies in Epidemiology (STROBE) reporting guideline. A sample of 642 deployed post-9/11 veterans from the Translational Research Center for Traumatic Brain Injury and Stress Disorders (TRACTS) study at VABHS were initially included in the study. Since 2009, the TRACTS research study has collected cognitive, psychological, and neurological functioning data on post-9/11 US veterans, with an emphasis on TBI and stress disorders.^32^ Participants repeat study procedures 1 year, 5 years, and 10 years after their initial study evaluation. The current study utilized data from baseline TRACTS visits, collected from 2009 to 2024. All participants provided written informed consent.

A total of 75 individuals were excluded, including 3 with impaired cognitive functioning, 3 with psychiatric instability, 4 with neurologic illness unrelated to TBI, 1 with insufficient information to determine TBI context, 4 with other concerns (eg, multidimensional outliers), 19 with history of moderate to severe TBIs, and 41 with suboptimal effort on performance validity testing (Medical Symptom Validity Test^33,34^).

### Boston Assessment of Traumatic Brain Injury–Lifetime (BAT-L)

The BAT-L is the widely accepted benchmark semistructured interview for retrospectively assessing head injuries throughout the lifespan. Thus, all head injuries assessed occurred prior to the study visit, and diagnoses were based on retrospective accounts of the head injury event. A doctorate-level psychologist administered the BAT-L^35^ to each participant using a forensic approach to determine the presence of TBI symptoms (ie, altered mental status, posttraumatic amnesia, and loss of consciousness). The detailed context and timeline of the injury are collected for each TBI, which includes additional information regarding the mechanism of injury and involvement of modifying factors (eg, substances or traumatic events). Clinicians assess potential TBIs during 3 distinct time epochs for each participant: predeployment, during military service, and postdeployment. All TBI diagnoses are graded for severity and confirmed by consensus of 3 or more doctorate-level psychologists.

### Peritraumatic Context Assessment

Researchers conducted a detailed retrospective review of BAT-L written records for every confirmed mTBI in the sample to assess the context in which each injury occurred. The Posttraumatic Diagnostic Scale (PDS-5)^36^ trauma types were used to guide the identification of possible peritraumatic contexts. Of note, peritraumatic contexts did not need to meet criteria for an A1 trauma, and a conservative approach was taken to all assessments; contexts were deemed not peritraumatic unless there was clear evidence to indicate it was peritraumatic. An important factor in determining peritraumatic context was the temporal parameters; researchers only considered the context at the time of the injury, not the time preceding or after the injury.

After all cases were independently reviewed, researchers completed a secondary review of all injuries that did not have a clear, identifiable context upon initial review, following inductive thematic analysis using PDS-5 trauma types for context.^37^ Each researcher independently reviewed the injuries with additional information, including interview audio recordings, and peritraumatic context was then determined as a consensus group. If insufficient information was available, those injuries were removed from the dataset (1 participant). Individuals who only experienced mTBI in nonperitraumatic contexts were included in the nonperitraumatic mTBI group, and those who experienced at least 1 mTBI within a peritraumatic context were included in the peritraumatic mTBI group.

### PTSD Severity

Participants were also administered the Clinician-Administered PTSD Scale–4th edition (CAPS-IV^38^) by a doctoral-level psychologist to assess PTSD severity the same day as the assessment of TBI history. PTSD symptoms were evaluated during 3 time epochs: (1) current (past month), (2) respective accounts of worst postdeployment symptoms (the period since returning from military deployment in which the veteran experienced their worst PTSD symptoms), and (3) retrospective account of predeployment symptoms (the period prior to military deployment in which the veteran experienced their worst PTSD symptoms). Predeployment CAPS-IV scores were only measured if the individual reported exposure to a trauma that would meet A1 criteria before enlisting in the military.

### Questionnaires

On the same day as TBI and PTSD assessments, participants completed multiple questionnaires assessing current symptoms. The Traumatic Life Events Questionnaire (TLEQ)^39^ assessed the history and frequency (0 = none; 6 = more than 5 times) of 22 potential traumatic event exposures. TLEQ total frequency score summed the frequency of all the traumatic events experienced (range, 0-132). The Neurobehavioral Symptom Inventory (NSI)^40^ measured 22 potential postconcussive symptoms experienced in the past 2 weeks on a 4-point scale (0 = none; 4 = very severe; range, 0-88). The World Health Organization Disability Assessment Schedule II (WHODAS)^41^ measured self-reported current disability across 36 activities over the past 30 days (0 = no difficulty; 5 = extreme disability; range, 0-180).

### Statistical Analysis

Differences between no TBI, nonperitraumatic mTBI, and peritraumatic mTBI groups in demographic, number of lifetime TBIs, and trauma exposure were examined with independent *t* tests with Fisher least significant difference (LSD) comparisons as post hoc analyses or χ^2^ tests, where appropriate. The significance threshold was set to *P* < .05. Analysis was conducted from January to October 2024 using SPSS version 29.0.1.0 (IBM).

#### PTSD Severity

Analyses of covariance (ANCOVAs) were performed to examine the association of mTBI with PTSD severity and CAPS-IV total score for current, postdeployment worst, and predeployment time points as separate dependent variables. Age, gender, education, number of lifetime TBIs, and TLEQ total frequency scores were included as covariates.

#### Postconcussive and Disability Outcomes

Differences in postconcussive symptoms (NSI total) and functional and disability self-ratings (WHODAS total) between mTBI groups were examined with ANCOVAs with age, gender, education, number of lifetime TBIs, and TLEQ total frequency scores as covariates. Follow-up analyses included CAPS-IV current score as an additional covariate to investigate if any observed associations were influenced by PTSD severity because PTSD is often associated with postconcussive symptoms and disability.

#### Supplemental Analyses

Differences in NSI and WHODAS subfactors between mTBI groups are within eAppendix 1 in Supplement 1. Additional analyses controlling for combat exposure and mTBI severity as covariates assessed whether differences in these factors influenced the results (eAppendix 2 in Supplement 1).

## Results

The sample consisted of 567 veterans (mean [SD] age, 33.72 [9.29] years; 507 men [89.4%]; mean [SD] years of education, 14.19 [2.16]), including 183 with no TBI, 189 with nonperitraumatic mTBI, and 195 with peritraumatic mTBI. Demographic, TBI, and trauma exposure–related variables between the TBI groups of the 567 veterans are summarized in Table 1. There was a significant difference in the distribution of gender between mTBI groups, with a greater proportion of women in the no TBI group (31 women [16.9%]) compared with the nonperitraumatic (14 women [7.4%]) and peritraumatic mTBI groups (15 women [7.7%]) (*P* = .003). The nonperitraumatic mTBI and peritraumatic mTBI groups did not significantly differ in their distribution of gender. The peritraumatic mTBI group reported more trauma exposure (mean [SD] TLEQ score, 22.93 [14.42]) than the nonperitraumatic mTBI group (mean [SD] TLEQ score, 17.38 [10.71]) and no TBI group (mean [SD] TLEQ score, 13.97 [10.74]) (*P* < .001) and the nonperitraumatic mTBI group reported more trauma exposure than the no TBI group (*P* < .001). Additionally, veterans in the peritraumatic mTBI group had more lifetime TBIs than the nonperitraumatic mTBI group (mean [SD] number of lifetime TBIs, 2.75 [2.38] vs 0.00 [0.00]; *P* < .001).

**Table 1.** Differences Between TBI Groups in Demographic, Lifetime TBI, and Trauma Exposure

Characteristic	Participants, Mean (SD) (N = 567)			*P* value
	No TBI (n = 183)	Nonperitraumatic mild TBI (n = 189)	Peritraumatic mild TBI (n = 195)	
Age, y	34.31 (9.57)	34.17 (9.58)	32.74 (8.28)	.19
Gender, No. (%)				
Men	152 (83.1)	175 (92.6)	180 (92.3)	.003^a^
Women	31 (16.9)	14 (7.4)	15 (7.7)	
Education, y^b^	14.43 (2.11)	14.21 (2.12)	13.95 (2.23)	.10
No. of lifetime TBIs	0	1.92 (1.70)	2.75 (2.38)	<.001^c^
TLEQ total frequency score	13.97 (10.74)	17.38 (10.71)	22.93 (14.92)	<.001^d^

Abbreviations: TBI, traumatic brain injury; TLEQ, Traumatic Life Events Questionnaire.

^a^ The no TBI group had a significantly greater proportion of women compared with the nonperitraumatic and peritraumatic mild TBI group. The nonperitraumatic and peritraumatic mild TBI groups did not significantly differ in their distribution of gender.

^b^ Education: 12 years = high school level; 14 years = associates degree or 2 years of college; 16 years = bachelor’s degree; and 20 years = doctorate degree.

^c^ The peritraumatic mild TBI group had significantly greater number of lifetime TBIs and greater lifetime trauma exposure (as measured by the TLEQ total frequency) than the nonperitraumatic mild TBI and no TBI groups.

^d^ The nonperitraumatic mild TBI group had a significantly greater number of lifetime TBIs and greater lifetime trauma exposure than the no TBI group.

### PTSD Severity

**Figure 1.**
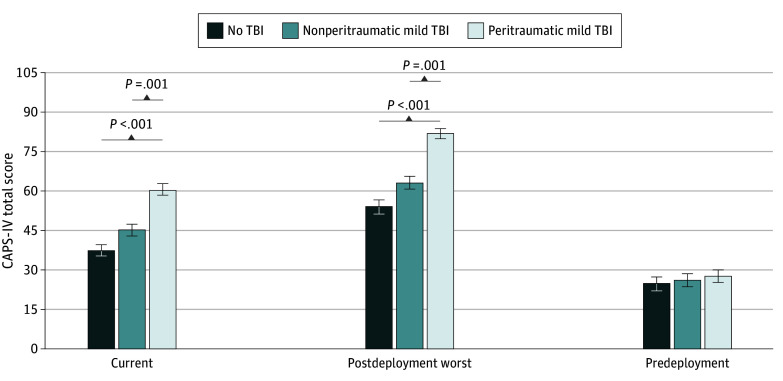
Differences in Current, Postdeployment, and Predeployment Posttraumatic Stress Disorder (PTSD) Severity Between Traumatic Brain Injury (TBI) Groups Actual mean scores of no TBI, nonperitraumatic mild TBI, and peritraumatic mild TBI in Clinician-Administered PTSD Scale–Fourth Edition scores (ie, PTSD severity) across different epochs: current, postdeployment worst time point, and predeployment worst time point. Error bars indicate SEs. Covariates included age, gender, education, total trauma exposure, and number of lifetime TBIs.

An ANCOVA revealed significant differences between groups on current (*F*_2,552_ = 8.45; *P* < .001; ηp^2^ = 0.030) and postdeployment worst CAPS-IV scores (*F*_2,530 _= 11.95; *P* < .001; ηp^2^ = 0.044) (Figure 1A and B). LSD comparisons revealed the peritraumatic mTBI group had significantly higher current CAPS-IV and postdeployment worst CAPS-IV scores than the no TBI group (current: mean difference, 12.24; 95% CI, 6.00 to 18.47; *P* < .001; postdeployment worst: mean difference, 16.65; 95% CI, 9.30 to 24.00; *P* < .001) and nonperitraumatic mTBI group (current: mean difference, 8.60; 95% CI, 3.36 to 13.85; *P* = .001; postdeployment worst: mean difference, 12.69; 95% CI, 6.44 to 18.93; *P* < .001), whereas the no TBI and nonperitraumatic mTBI groups did not significantly differ in current (mean difference, 3.63; 95% CI, −2.05 to 9.32; *P* = .21) or postdeployment worst CAPS-IV score (mean difference, 3.96; 95% CI, −2.86 to 10.79; *P* = .26). There were no significant differences between mTBI groups in CAPS-IV score during the predeployment epoch (*F*_2,303_ = 0.550; *P* = .58; ηp^2^ = 0.004) (Figure 1C).

### Postconcussive and Disability Outcomes

**Figure 2.**
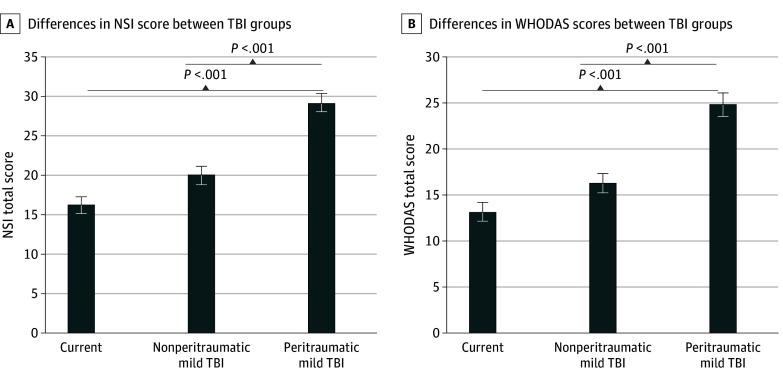
Differences in Neurobehavior Symptoms Index (NSI) Score and World Health Organization Disability Assessment Schedule (WHODAS) Total Score Between Traumatic Brain Injury (TBI) Groups A, Actual mean scores of no TBI, nonperitraumatic mild TBI, and peritraumatic mild TBI in NSI total score (ie, postconcussive symptoms). B, Actual mean scores of no TBI, nonperitraumatic mild TBI, and peritraumatic mild TBI in WHODAS total score (ie, disability). Error bars indicate SEs. Covariates included age, gender, education, total trauma exposure, and number of lifetime TBIs.

An ANCOVA revealed significant differences between TBI groups in the NSI total (*F*_2,533_ = 11.09; *P* < .001; ηp^2^ = 0.040) and WHODAS total scores (*F*_2,527_ = 11.13; *P* < .001; ηp^2^ = 0.041) (Figure 2A and B). LSD comparisons indicated the peritraumatic mTBI group had significantly higher NSI symptoms and WHODAS disability scores than the no TBI group (NSI: mean difference, 8.12; 95% CI, 4.38 to 11.85; *P* < .001; disability: mean difference; 7.77; 95% CI, 4.18 to 11.36; *P* < .001) and nonperitraumatic mTBI group (NSI: mean difference, 6.38; 95% CI, 3.18 to 9.58; *P* < .001; disability: mean difference, 6.22; 95% CI, 3.14 to 9.30; *P* < .001). There were no significant differences between the no TBI and nonperitraumatic mTBI groups in NSI total (mean difference, 1.74; 95% CI, −1.70 to 5.18; *P* = .32) and WHODAS total scores (mean difference, 1.55; 95% CI, −1.75 to 4.85; *P* = .36). These results remained similar after additionally controlling for current CAPS-IV score (eAppendix 1 in Supplement 1). Outcome means and adjusted estimated means for covariates are presented in Table 2 and eAppendix 3 in Supplement 1.

**Table 2.** Actual and Estimated Means Within Mild TBI Groups in PTSD Severity, Postconcussive Symptoms, and Disability

Mean type and outcome measure	No TBI (n = 183)	Nonperitraumatic mild TBI (n = 189)	Peritraumatic mild TBI (n = 195)
Actual, mean (SE)			
CAPS-IV			
Current	37.47 (28.02)	45.30 (28.22)	60.31 (27.48)^a^
Postdeployment worst	54.04 (33.21)	63.10 (33.19)	81.99 (27.93)^a^
Predeployment	24.91 (23.71)	26.07 (26.19)	27.54 (25.93)^a^
NSI total	16.19 (14.79)	20.06 (15.33)	29.09 (16.66)^a^
WHODAS total	13.10 (13.56)	16.39 (14.15)	24.85 (15.84)^a^
Estimated, mean (SE) [95% CI]^b^			
CAPS-IV			
Current	42.56 (2.14) [38.35-46.76]	46.19 (1.86) [42.54-49.84]	54.59 (1.97) [50.92-58.66]^a^
Postdeployment worst	59.69 (2.54) [54.70-64.68]	63.66 (2.25) [59.23-68.08]	76.34 (2.32) [71.69-80.89]^a^
Predeployment	25.28 (2.77) [19.82-30.74]	28.25 (2.35) [23.63-32.88]	25.15 (2.48) [20.28-30.03]^a^
NSI total	18.51 (1.26) [16.03-20.99]	20.25 (1.15) [18.00-22.20)]	26.63 (1.20) [24.27-28.99]^a^
WHODAS total	15.011 (1.21) [12.64-17.39]	16.56 (1.10) [14.40-18.72]	22.78 (1.16) [20.50-25.06]^a^

Abbreviations: CAPS-IV, Clinician-Administered PTSD Scale–Fourth Edition; NSI, Neurobehavioral Symptom Inventory; PTSD, posttraumatic stress disorder; TBI, traumatic brain injury; WHODAS, World Health Organization Disability Assessment Schedule II.

^a^ The peritraumatic mild TBI group had significantly greater PTSD severity, postconcussive symptoms, and disability than the nonperitraumatic mild TBI and no TBI groups. The nonperitraumatic mild TBI and no TBI groups did not significantly differ in any outcome measure.

^b^ Covariates for the adjusted estimated means included age, gender, education, total trauma exposure, and number of lifetime TBIs. See eAppendix 4 in Supplement 1 for mean differences and 95% CIs between groups.

### Supplemental Analyses

After additionally controlling for combat exposure and mTBI severity, results remained similar to the main analyses, with the peritraumatic mTBI group showing significantly greater postdeployment worst PTSD severity, postconcussive symptoms, and disability than the nonperitraumatic mTBI and no TBI groups. There were no significant differences between the nonperitraumatic mTBI and no TBI groups (eAppendix 2 and eAppendix 4 in Supplement 1).

## Discussion

This cohort study is the first, to our knowledge, to examine the unique consequences associated with mTBI sustained in a peritraumatic context compared with those that occurred in nonperitraumatic contexts. Veterans with a history of peritraumatic mTBI demonstrated poorer long-term outcomes, including greater PTSD severity, postconcussive symptoms, and disability compared with veterans with a history of nonperitraumatic mTBI or with no TBI history. In contrast, and perhaps most critically, the nonperitraumatic mTBI and no TBI groups did not significantly differ in any study outcomes, which indicated that mTBI was only associated with negative long-term outcomes when it co-occurred with a traumatic event exposure. Additionally, the differences in postconcussive symptoms and disability observed within the peritraumatic mTBI group remained significant even when controlling for PTSD severity. These results demonstrate that having a comorbid history of mTBI and PTSD was not in itself associated with deficits but that sustaining an mTBI within a peritraumatic context was associated with enduring consequences across several measures.

Specifically, these findings show that peritraumatic mTBI, but not nonperitraumatic mTBI, displayed greater PTSD severity, suggesting when mTBI occurs within the sensitive period surrounding a traumatic event, it may be associated with an increased susceptibility to the development of PTSD. Previous studies have suggested that 10-point differences in the CAPS-IV is a clinically significant difference.^42^ In the present study, the peritraumatic TBI group had PTSD severity mean scores that were around or above this 10-point difference compared with the other groups, highlighting the important clinical relevance of these findings. Additionally, the current results demonstrated that peritraumatic mTBI, but not nonperitraumatic mTBI, showed greater postconcussive symptoms and disability. The supplementary analyses (see eAppendix 4 in Supplement 1) reiterate these findings and show that, when using clinical cutoff points for disability, individuals with peritraumatic mTBI were more likely to meet criteria for disability compared with nonperitraumatic and no TBI groups, further supporting the clinical importance of these results. Taken together, these novel findings may provide a potential explanation for the array of contradictory data concerning the long-standing effects of mTBI.

As previously discussed, there are 3 mutually contradictory frameworks that have been used to describe poor long-term outcomes following mTBI. The first and second frameworks assert opposite views, with the former attributing all long-term consequences to mTBI without consideration of common comorbidities and the latter attributing all long-term consequences to comorbidities, such as PTSD, rather than the mTBI itself. The third approach contradicts both the first and second, suggesting mTBI and PTSD are independent conditions that impart separate, cumulative chronic effects on poor long-term outcomes. However, these approaches consider mTBI and PTSD as isolated conditions that occur independently without acknowledging that oftentimes the exact circumstances that cause mTBIs also result in psychological trauma leading to PTSD.

To reconcile these discrepancies, we propose a new hypothetical framework, the peritraumatic scaffolding effect. This framework suggests that while mTBI on its own may not directly cause chronic psychiatric, postconcussive, and functional problems, the acute impact of mTBI could catalyze or scaffold PTSD-related psychopathological symptoms, which are then associated with poorer long-term outcomes. This proposed scaffolding framework hypothesizes that mTBI may temporarily set the circumstances to promote the formation of PTSD symptoms, allowing trauma and PTSD to exert mounting negative consequences on long-term postconcussive symptoms and functional abilities. Thus, it argues that the mTBI alone is not associated with these enduring deficits, but rather poor long-term outcomes may be associated with the acute amplification of trauma-related effects that the co-occurring mTBI could be scaffolding at the time of injury. Unlike the 3 previous approaches that treat mTBI and PTSD as separate entities, these data suggest that mTBI and trauma may be intertwined, and that peritraumatic mTBI is associated with different risks and worse long-term outcomes than an mTBI occurring outside of this peritraumatic context.

Although the present study did not provide data that directly supports the mechanisms of this proposed scaffolding hypothesis, the existing PTSD and acute mTBI literature cites several processes through which a peritraumatic mTBI may increase one’s risk for PTSD formation. One potential mechanism may be mediated by an individual’s reaction to a traumatic event. Increasing evidence suggests cognitive appraisal processes and dissociative responses following a psychologically traumatic event were among the best predictors of PTSD symptom formation.^43^ The acute disruption in attention and information processing caused by an mTBI may temporarily alter the cognitive appraisal processes (eg, dissociating or not elaborating on the contextual elements) that are critical to PTSD resilience.^30^ Additionally, the effects of acute mTBI on PTSD may be physiological or neurobiological, such as through autonomic dysregulation during the acute period of mTBI^44^ to increase the likelihood of developing PTSD symptoms.^45,46^ Finally, mTBI may temporarily disrupt PTSD-related structural and functional neural circuitry, including the limbic system,^47,48^ hindering the regulation of certain emotions and behaviors.^49,50^ Future research should examine different mechanisms through which peritraumatic mTBI may engender poorer long-term outcomes, particularly the development of PTSD.

This research attempted to unify and reconcile the 3 empirical approaches used in the mTBI literature to date and the resultant explanatory frameworks describing the long-term consequences of mTBI. However, because the current study is the first, to our knowledge, to demonstrate a potential distinction between peritraumatic and nonperitraumatic mTBI, this novel framework raises many empirical questions, particularly regarding how to characterize the critical temporal window of these effects. For example, it is unknown how the order of the mTBI and trauma exposure (eg, if the mTBI precedes or succeeds the traumatic event) or how the duration and/or severity of TBI symptoms and trauma exposure may influence the potential scaffolding effects and subsequent consequences. To our knowledge, the data needed to answer such questions are not readily available and will need to be collected in future studies.

### Limitations

There are multiple limitations to the present study. First, the study population is largely White men, limiting the generalizability of the findings, particularly to civilian contexts. Second, peritraumatic mTBI data was evaluated retrospectively via detailed BAT-L record review and consensus coding. In the future, a more accurate conceptualization of a peritraumatic context would be best captured immediately after injury. Third, self-report data can be subject to recall bias; however, excluding individuals who failed a sensitive effort measure (ie, Medical Symptom Validity Test) increases the validity of these data. Many previous studies examining the long-term effects of mTBI neglect measuring and/or precluding individuals who give suboptimal effort within their samples, which may exacerbate biases and lead to inaccurate data.^34^ Fourth, although PTSD symptom severity was considered at multiple time points, these data are cross-sectional in design. Research with longitudinal data are needed to establish causal relationships between peritraumatic mTBI and decline over time.

## Conclusions

This cohort study of 567 veterans is the first, to our knowledge, to demonstrate that mTBIs sustained in a peritraumatic context were associated with worse long-term outcomes, including increased PTSD severity, enduring postconcussive symptoms, and greater disability. Notably, individuals with a history of mTBI in a nonperitraumatic context did not significantly differ from those with no TBI history on any outcome measure. The findings from the current study have important theoretical and clinical implications, providing a possible explanation for the decades of contradictory results published in the literature on long-term outcomes of mTBI. The proposed comprehensive explanatory framework, peritraumatic scaffolding, suggests that mTBI occurring in an emotionally traumatic context may be associated with temporary disruptions of psychological and/or physiological processes that are critical to remaining resistant to the development of PTSD and other long-term consequences.

## Notes

Supplement 1. **eAppendix 1.** Subfactors **eAppendix 2.** Supplementary Results **eAppendix 3.** Supplementary Tables **eAppendix 4.** Supplementary Analysis

Supplement 2.
Data Sharing Statement
