# Supplementary material for: Peritraumatic Context and Long-Term Outcomes of Concussion
Source: JAMA Netw Open. 2025 Jan 22;8(1):e2455622. doi: 10.1001/jamanetworkopen.2024.55622 (PMC11755194; doi:10.1001/jamanetworkopen.2024.55622)
Supplement: Supplement 1. — eAppendix 1. Subfactors eAppendix 2. Supplementary Results eAppendix 3. Supplementary Tables eAppendix 4. Supplementary Analysis [file jamanetwopen-e2455622-s001.pdf]

Supplemental Online Content

Van Etten EJ, Knight AR, Colaizzi TA, et al. Peritraumatic context and long-term outcomes of concussion. *JAMA Netw. Open*. 2025;8(1):e2455622.
doi:10.1001/jamanetworkopen.2024.55622

eMaterials 1. Subfactors
eMaterials 2. Supplementary Results
eMaterials 3. Supplementary Tables
eMaterials 4. Supplementary Analysis

This supplemental material has been provided by the authors to give readers additional information about their work.

© 2025 Van Etten EJ et al. *JAMA Network Open*.

eMaterials 1. Subfactors

Postconcussive Subfactors:

The Neurobehavioral Symptom Inventory (NSI; Cicerone & Kalmar, 1995; King et al., 2012) measures 22 potential post-concussive symptoms that may emerge after a TBI (e.g., poor coordination; forgetfulness) with ratings of how often and severe each symptom bothers the individual (0 = Rarely if ever-present; not a problem at all to 4 = Almost always present and I have been unable to perform at work, school or home due to this problem). The NSI consists of four subfactors, including vestibular (e.g., dizziness, balance and coordination problems), somatic (e.g., headaches, nausea, vision/hearing issues), cognitive (e.g., concentration and memory difficulties), and affective (e.g., fatigue, anxiety/depression, and irritability complaints) factors.

Disability Subfactors:

The WHO Disability Assessment Schedule (WHODAS; WHO, 2010) is a 36-item questionnaire measuring health and disability across multiple activities over the past 30 days, with higher scores indicating greater disability (1 = no difficulties with an activity; 5 = extreme difficulties or inability to do the activity). There are 6 subfactors within the WHODAS, which examine different domains of functioning, including: communication (understanding and communicating), mobility (moving and getting around), self-care (hygiene, dressing, & eating), getting along (interacting with other people), life and occupational activities (domestic responsibilities & work and/or school difficulties), and participating socially (joining in community activities).

Results

Post-concussive Symptom and Disability Total:

The following analyses are examining differences between TBI groups on post-concussive symptom and disability total (similar to main manuscript analyses) while additionally

© 2025 Van Etten EJ et al. *JAMA Network Open*.

controlling for current PTSD severity. An ANCOVA additionally controlling for current CAPS-IV score (along with age, gender, education, number of lifetime TBIs and TLEQ total frequency) revealed significant differences between groups in NSI total score (F(2, 525) = 3.13, p = .028; η_p^2 = .014). LSD comparisons showed the peritraumatic mTBI group had significantly higher NSI total scores than no-TBI (p = .017) and non-peritraumatic mTBI (p = .022) groups, but there were no significant differences in NSI total scores between no-TBI and non-peritraumatic mTBI groups (p = .642).

A follow-up ANCOVA additionally controlling for current CAPS-IV score (along with age, gender, education, number of lifetime TBIs, and TLEQ total frequency) revealed significant differences between TBI groups in total WHODAS score (F(2, 519) = 3.52, p = .022; η_p^2 = .015). LSD comparisons showed the peritraumatic mTBI group had significantly higher WHODAS total scores than no-TBI (p = .014) and non-peritraumatic mTBI (p = .018) groups; there were no significant differences in WHODAS total scores between no-TBI and non-peritraumatic mTBI groups (p = .634).

Post-concussive Symptom Subfactors:

After controlling for age, gender, education, number of lifetime TBI's, and TLEQ total frequency an ANCOVA revealed significant differences between TBI groups in all post-concussive subfactors (vestibular, somatic, cognitive, and affective) of the NSI. LSD comparisons showed that these subfactors followed a similar pattern in which the peritraumatic TBI group reported greater post-concussive symptoms in these subfactors than the no-TBI and non-peritraumatic TBI groups (p's < .05), whereas the no-TBI and non-peritraumatic TBI groups did not significantly differ (p's > .05).

After additionally controlling for CAPS-IV current scores, an ANCOVA revealed significant differences between TBI groups in the vestibular (p = .003), somatic (p = .032), and cognitive (p = .005) subfactors of the NSI. LSD comparisons showed that the vestibular,

© 2025 Van Etten EJ et al. *JAMA Network Open*.

cognitive, and somatic subfactors all followed a similar pattern in which the peritraumatic mTBI group reported greater post-concussive symptoms in these subfactors than the no-TBI and non-peritraumatic TBI groups (*p*'s < .05), whereas the no-TBI and non-peritraumatic mTBI groups did not significantly differ (*p*'s > .05). There were no significant differences between TBI groups in the affective subfactor.

<u>Disability Subfactors</u>:

After controlling for age, gender, education, number of lifetime TBI's, and TLEQ total frequency an ANCOVA revealed significant differences between TBI groups in the communication, mobility, getting along, life and work activity, and participating socially subfactors of the WHODAS. LSD comparisons showed, these subfactors followed a similar pattern in which the peritraumatic TBI group showed greater disability in these subfactors than the no-TBI and non-peritraumatic TBI groups (*p*'s < .05), whereas the no-TBI and non-peritraumatic TBI groups did not significantly differ (*p*'s > .05). There were no significant differences between TBI groups in the self-care subfactor.

After additionally controlling for CAPS-IV current scores, an ANCOVA revealed significant differences between TBI groups only in the communication subfactor (*p* = .003), with LSD comparison showed the peritraumatic TBI group showed had greater disability in the communication subfactor than the no-TBI (*p* = .002) and non-peritraumatic mTBI groups (*p* = .004). The no-TBI and non-peritraumatic mTBI groups did not significantly differ on this subfactor (*p*'s = .825). There were no significant differences between TBI groups in the mobility, getting along, life and work activity, participating socially, and self-care subfactors.

© 2025 Van Etten EJ et al. *JAMA Network Open*.

eMaterials 2. Supplementary Results

Combat Exposure:

The Deployment Risk and Resilience Inventory (DRRI; King et al., 2006), assesses exposure to combat-related experiences, such as being attacked (e.g., encountering an explosive device), or going on patrols that potentially involve combat experiences. Participants rate how often they encountered each experience on a 6-point likert scale (from 1 = never and 6 = daily or almost daily); thus, higher scores indicate greater exposure to combat-related experiences on deployment.

Traumatic Brain Injury Severity:

A doctorate-level psychologist administered the BAT-L (Fortier et al., 2014) to each participant. The BAT-L is a gold standard semi-structured interview designed to retrospectively assess military and civilian head injuries throughout the lifespan using a forensic approach. TBI diagnosis and severity are determined by the timeline and presence of TBI symptoms (i.e., altered mental status (AMS), posttraumatic amnesia, and loss of consciousness). For an estimate of severity of TBI, the aggregate amount of AMS was used.

Results

The following analyses are the same analyses as presented in the main manuscript while additionally controlling for combat exposure and mTBI severity as covariates to assess whether differences in these factors influenced our results.

CAPS-IV:

An ANCOVA controlling for age, gender, education, number of lifetime TBIs, TLEQ total frequency, combat exposure, and TBI severity revealed marginal differences between groups on current CAPS-IV score ($F(2, 525) = 2.84$, $p = .09$, $\eta_p^2 = .011$). LSD comparisons showed the peritraumatic mTBI group had greater PTSD severity than the no-TBI ($p = .021$), but marginal differences compared to the non-peritraumatic mTBI groups ($p = .073$). There were no

© 2025 Van Etten EJ et al. *JAMA Network Open*.

significant differences between the no-TBI and non-peritraumatic mTBI groups in current CAPS-IV scores (p = .389).

An ANCOVA controlling for age, gender, education, number of lifetime TBIs, TLEQ total frequency, combat exposure, and TBI severity revealed significant differences between groups in post-deployment worst CAPS-IV scores (F(2, 505) = 6.16, p = .002, η_p^2= .024). Similar to our results in the main manuscript, LSD comparisons showed the peritraumatic mTBI group had significantly higher post-deployment worst CAPS-IV scores than the no-TBI (p = .002) and non-peritraumatic mTBI groups (p = .003). There were no significant differences between the no-TBI and non-peritraumatic mTBI groups in post-deployment worst CAPS-IV scores (p = .525).

An ANCOVA controlling for the age, gender, education, number of lifetime TBIs, TLEQ total frequency, combat exposure, and TBI severity there were still no significant differences between mTBI groups in CAPS-IV scores during the pre-deployment epoch (F(2, 283) = .253, p = .777, η_p^2= .002).

Post-concussive Symptom Total:

After controlling for age, gender, education, number of lifetime TBI's, TLEQ total frequency, CAPS-IV current score, combat exposure, and TBI severity an ANCOVA revealed significant differences between TBI groups in the NSI total score (F(2, 519) = 3.62, p = .027, η_p^2= .014). Similar to our results in the main manuscript, LSD comparisons showed the peritraumatic mTBI group had significantly higher postconcussive symptoms than the no-TBI (p = .016) and non-peritraumatic mTBI groups (p = .019). There were no significant differences between the no-TBI and non-peritraumatic mTBI groups in post-concussive symptoms (p = .666).

Disability Total:

After controlling for age, gender, education, number of lifetime TBI's, TLEQ total frequency, CAPS-IV current score, combat exposure, and TBI severity an ANCOVA revealed significant differences between TBI groups in WHODAS total score (F(2, 513) = 4.00, p = .019,

© 2025 Van Etten EJ et al. *JAMA Network Open*.

η_p^2= .016). Similar to our results in the main manuscript, LSD comparisons showed the peritraumatic mTBI group had significantly greater disability than the no-TBI (p = .012) and non-peritraumatic mTBI groups (p = .013). There were no significant differences between the no-TBI and non-peritraumatic mTBI groups in functional abilities (p = .686).

© 2025 Van Etten EJ et al. *JAMA Network Open*.

eMaterials 3. Supplementary Tables

Supplemental Table 1. Differences in current PTSD severity between mTBI groups.

Comparison	Mean difference	95% confidence interval for difference
Peritraumatic mTBI compared to no-TBI	12.24	6.00, 18.47
Peritraumatic mTBI compared to non-peritraumatic mTBI	8.60	3.36, 13.85
Non-peritraumatic mTBI compared to no-TBI	3.63	-2.05, 9.32

Supplemental Table 2. Differences in post-deployment worst PTSD severity between mTBI groups.

Comparison	Mean difference	95% confidence interval for difference
Peritraumatic mTBI compared to no-TBI	16.65	9.30, 24.00
Peritraumatic mTBI compared to non-peritraumatic mTBI	12.69	6.44, 18.93
Non-peritraumatic mTBI compared to no-TBI	3.96	-2.86, 10.79

Supplemental Table 3. Differences in pre-deployment PTSD severity between mTBI groups.

Comparison	Mean difference	95% confidence interval for difference
Peritraumatic mTBI compared to no-TBI	-.130	-8.10, 7.84
Peritraumatic mTBI compared to non-peritraumatic mTBI	-3.10	-9.79, 3.58
Non-peritraumatic mTBI compared to no-TBI	2.97	-4.30, 10.24

Supplemental Table 4. Differences in post-concussive symptoms between mTBI groups.

Comparison	Mean difference	95% confidence interval for difference
Peritraumatic mTBI compared to no-TBI	8.12	4.38, 11.85
Peritraumatic mTBI compared to non-peritraumatic mTBI	6.38	3.18, 9.58
Non-peritraumatic mTBI compared to no-TBI	1.74	-1.70, 5.18

Supplemental Table 5. Differences in disability between mTBI groups.

Comparison	Mean difference	95% confidence interval for difference
Peritraumatic mTBI compared to no-TBI	7.70	4.18, 11.36
Peritraumatic mTBI compared to non-peritraumatic mTBI	6.22	3.14, 9.30
Non-peritraumatic mTBI compared to no-TBI	1.55	-1.75, 4.85

© 2025 Van Etten EJ et al. *JAMA Network Open*.

eMaterials 4. Supplementary Analysis

In an effort to provide more clinically-relevant information about our disability measure, we used clinical cut-offs based on prior literature (Bovin et al., 2019) for the WHODAS measure. Bovin and colleagues (2019) found that a cut-off score of 32 indicates disability within a PTSD sample. We used this cut-off score within our sample and conducted a binary logistic regression to predict the odds ratio of meeting disability, comparing the peritraumatic mTBI group to the no-TBI and non-peritraumatic groups with age, gender, education, number of lifetime TBIs, and total trauma exposure as covariates. The results replicated our original main analyses with continuous variables and indicated that the peritraumatic TBI group has significantly greater odds for meeting criteria for this disability cut-off (OR: 3.723 [CI: 1.91, 7.27] than the non-peritraumatic TBI and no-TBI groups. The non-peritraumatic TBI and no TBI groups did not significantly differ in their odds for meeting this criteria for disability cut-off (OR: 1.31 [CI: .657, 2.62]. However, the number of individuals that met criteria for disability were low, with the no TBI group only having 19 people meeting criteria for disability (out of 178 total), the non-peritraumatic TBI group having 26 people (out of 172 total), and the peritraumatic TBI group having 68 people meeting criteria (out of 179 total). Thus, although these analyses closely follow and reiterate our main analyses, they should be interpreted with caution.

© 2025 Van Etten EJ et al. *JAMA Network Open*.
