# Supplementary material for: Peritraumatic Context and Long-Term Outcomes of Concussion
Source: JAMA Netw Open. 2025 Jan 22;8(1):e2455622. doi: 10.1001/jamanetworkopen.2024.55622 (PMC11755194; doi:10.1001/jamanetworkopen.2024.55622)
Supplement: Supplement 2. — Data Sharing Statement [file jamanetwopen-e2455622-s002.pdf]

Data Sharing Statement

Van Etten. Peritraumatic Context and Long-Term Outcomes of Concussion. *JAMA Netw Open*. Published January 22, 2025. doi:10.1001/jamanetworkopen.2024.55622

Data
Data available: No

Additional Information
Explanation for why data not available: The data is owned by US Department of Veterans Affairs and therefore will not be made publicly available. Inquiries to access data can be made to the Translational Research Center for TBI and Stress Disorders (TRACTS) through the corresponding author.
